# Does calf-mother contact during heat stress period affect physiology and performance in buffaloes?

**DOI:** 10.5713/ab.23.0382

**Published:** 2024-02-22

**Authors:** Nripendra Pratap Singh, Madan Lal Kamboj

**Affiliations:** 1 Livestock Production Management Division, ICAR-National Dairy Research Institute (NDRI), Karnal-132001, India

**Keywords:** Fenceline Calf Contact, Fogger-fan, Heat Stress, Milk Yield, Murrah Buffalo

## Abstract

**Objective:**

Objective of the study was to reduce heat stress in Murrah buffaloes and maintain their milk production and other vital functions during heat stress.

**Methods:**

A total of 21 dyads of calf-mother Murrah buffalo were selected for the study and equally divided in 3 treatment groups. First treatment group was restricted calf contact (RCC), second treatment group was fence line calf contact (FCC) and third treatment groups fence line calf contact and heat stress protection (FCC-HSP [time-controlled fan-fogger system] in the shed). Present study was conducted from April to mid-September 2021.

**Results:**

Maximum temperature and temperature humidity index in FCC-HSP shed were significantly (p<0.05) lower than that in FCC and RCC shed. Higher (p<0.05) mean daily milk yield in both the treatment groups FCC (10.36±0.30) and FCC-HSP (10.97±0.31) than RCC (8.29±0.41) was recorded. Though no significant difference between FCC and FCC-HSP in daily milk yield but FCC-HSP yielded 600 gm more milk than FCC. Pulse rate (PR) and respiration rate (RR) were lowest in FCC-HSP followed by FCC and RCC, respectively. Cortisol and prolactin levels were lower (p<0.05) in FCC-HSP followed by FCC and RCC, respectively.

**Conclusion:**

Hence, FCC along with heat stress ameliorative measures helped the buffaloes to be free of stress and maintain milk yield during heat stress period of the year in tropical conditions.

## INTRODUCTION

Due to environmental and nutritional challenges, livestock productivity in tropical areas is often lower than in temperate climates. Predictions suggest that the temperature is expected to increase by 1.8°C to 4°C by the year 2100 [1]. The Intergovernmental Panel on Climate Change (IPCC) has additionally pointed out that developing countries such as India are at greater risk of experiencing extreme climate events due to their heavy reliance on climate-sensitive sectors like agriculture and related industries.

Buffaloes are prone to heat stress because of their heightened vulnerability due to their dark skin color, sparser hair, and lower density of sweat glands on skin [2]. They have a thicker epidermis which lowers their capacity for cutaneous evaporation making them less able to expel extra metabolic heat and more vulnerable to heat stress [3]. Major environmental factors affecting buffaloes in India are air temperature, relative humidity, solar radiation and temperature humidity index (THI). All these environmental elements lead to heat stress, which is defined as a situation where a combination of environmental variables producing conditions that are higher than the temperature range of the animal’s thermoneutral zone. Various studies have shown that rapid fluctuations in temperature (either a significant increase or decrease in maximum/minimum temperature) in the summer months can intensify the stress on buffaloes, negatively impacting their reproductive and productive capabilities, ultimately resulting in significant financial losses [4].

Numerous biological functions like endocrine functions [3] are disturbed in buffaloes when they are exposed to heat stress leading to reduction in productive and reproductive performance [5]. Decline in the daily milk and overall milk output, and a shortened period of lactation are common during the summer months in buffaloes as a result of the stress they undergo during this season [6,7]. Heat stress results in a rise in temperature and respiratory rate [8], and increased cortisol levels in buffaloes which makes them more prone to loss in production. Welfare status of buffaloes is highly compromised due to heat stress and sometime severe heat stress also leads to death. In addition to its direct impact on livestock, climate change also exerts indirect effects that significantly impede livestock production, with a key indirect effect being the diminished accessibility of both feed and water for the livestock [9].

Under natural settings calf-mother contact soon after birth develops into a preferred reciprocal social relationship between a mother and the newborn leading to an emotional attachment between the two that endures brief separations [10]. The aspects of this relationship include nourishment, comfort, safety, allogrooming, synchronizing activities, resting in contact, and maintaining proximity which acts as a social buffer and has a relaxing impact on both mother and calf. However, this social interaction is broken in the commercial and organized dairy farming system when shortly after the birth calves are weaned from their mothers. Outcome of this is increased stress, decreased productivity, and abnormal behaviour in both mothers and calves [11]. Allowing fence line interaction between the dam and the offspring lowers the stress reaction to weaning compared to remote separation [12].

Under modern husbandry situations, the separation of cows and calves has been reported to have several adverse consequences including elevated stress levels, and decreased performance in both mothers and their calves in cattle [13] and buffaloes [14]. Buffaloes are known to have greater bonding with their calves and the mother-calf separation may have much more negative consequences than in other species. It is observed that higher daily milk yield is obtained in natural suckled buffaloes than weaned buffaloes [15].

Various methods have been developed to reduce heat stress on buffaloes so that the production of buffaloes is not hampered. These methods basically work on increasing heat dissipation and reducing skin temperature thereby providing comfort to buffaloes. Provisions like ceiling fans, spray cooling, misters, foggers, and their combinations like sprinkler with a fan cooling system are widely used to reduce heat stress in buffaloes [16]. Another method to reduce heat stress in buffaloes is wallowing, which is also considered as their natural behaviour [17]. Of the all the methods evaporative cooling has shown good result in reducing the THI thereby maintaining production of buffaloes [18]. When a fogger system is paired with forced air movement (fan) it accelerates body heat loss by up to three or four times [19], this paired system works on the principle of an evaporative cooling system. Further, an evaporative cooling system helps to boost milk production (8 to 13 kg/d) and animal feed intake (7% to 10%) while lowering body temperature (by 0.2°C to 0.5°C) and an increase in respiratory rate by around 20% to 25% [20].

In view of this discussion, the aim of this research was to study the effect of fence line mother-calf contact and suckling in conjunction with heat stress amelioration measures during summer season on the production performance and physiological measures in freshly calved Murrah buffaloes.

## MATERIALS AND METHODS

### Location of experiment and climatic condition

The study was conducted at the Livestock Research Centre (LRC), ICAR-NDRI, Karnal, situated at 29° 42′ 20″ N Latitude and 76° 58′ 52.5″ E Longitude, at an altitude of 247 m above mean of sea level. The maximum ambient temperature ranges from 42°C to 46°C in summer and 2°C to 5°C in winter with diurnal variation of 16°C to 22°C. The average rainfall in the region is around 650 mm.

### Experimental animals

The study was conducted during summer from April to mid-September. For the study, a total of 21 advanced pregnant buffaloes (parity 1 to 5) belonging to Murrah breed were selected from the institute’s buffalo herd. The buffaloes were moved to the calving pen 15 days prior to their anticipated calving date. Following a successful calving, the buffalo and calf pairs were divided into three groups (n = 7 each), ensuring that the groups had similar parity levels (3.43±0.43). In treatment group I, the buffaloes were allowed to suckle their calves’ colostrum and then milked twice daily and after milking were separated from their calves. They were kept in a loose house and were not provided with additional heat stress protection measures. This was termed as restricted calf contact (RCC). In group II, the buffaloes were housed in a fence line contact round-the-clock with their calves and were allowed to suckle their calves twice daily after morning and evening milking as in case of group I. This group was also housed in a loose house with no specific heat stress protection measures. This was referred to as fence line calf contact (FCC). In group III, the buffaloes had fence line contact with their calves round-the-clock and were allowed to suckle their calves twice daily as in case of group II, but were provided with a time controlled fogging and fanning system as a protection measure from heat stress. This was referred to as fenceline calf contact-heat stress protection (FCC-HSP) group.

### Housing and feeding of experimental animals

#### Housing

Before 15 days of the calving date, advanced pregnant animals were transferred to separate calving pens. Calving pens had both open and covered area adjacent to each other, a total floor space of 12 m^2^ was provided in each pen and floor was made of concrete so rubber mattress were provided on floor to avoid injuries. Six days post calving, the buffaloes were shifted to the experimental shed with covered and open areas adjoining each other. The experimental shed of FCC and FCC-HSP group had a fence line barrier which was constructed throughout the length of the shed including the covered and open area. It was constructed from galvanized iron pipes (0.0254 meter) and stood 1.524 m from the ground. Wire mesh (0.01×0.01 m) was used to provide for enough visual, aural, olfactory, and minimal touch interaction. Further in FCC-HSP group shed foggers and fans were installed in covered area. The automated time-based fogger cooling devices were installed at an 2.438 m height in a covered space. Two side wall fans (0.9144 m) facing in same direction at distance of 6 m from each other, blowing air at 10,000 to 11,000 cfm with a maximum throw distance of 9 m were installed. Cross foggers at an angle of 90° with four outlets, fogging 85 micron droplets at 22 liters per hour. The time for fogger and fan was adjusted as 45 seconds fogging and rest fanning in every 5 minutes from 11:00 to 16:00 h.

#### Feeding

All buffaloes were offered ration according to the ICAR (2013) [21] buffalo feeding standards. Seasonal farm-grown green fodder and dry roughage were given to the buffaloes *ad libitum*. The concentrate mixture composition is presented in the Table 1. *Ad libitum* clean and fresh water was provided. Buffaloes were milked by hand twice daily.

### Ethical approval

The study was authorized and carried out in accordance with the approved guidelines of the Institutional Animal Ethics Committee (IAEC). This committee operates in accordance with Article 13 of the regulations set forth by the Committee for Control and Supervision of Experiments on Animals (CCSEA), as stipulated by the Government of India (IAEC Approval No. 46-IAEC-20–8).

### Recording of climatic variables and temperature humidity index

To interpret the THI of each shed Zeal (UK) dry-wet bulb thermometers were used to record dry and wet bulb temperatures between 2.30 P.M to 3.00 P.M daily during experimental period. Temperature humidity index was calculated using the formula of NRC [22].


THI=0.72 (Tdb+Twb)+40.6

Where, Tdb, dry bulb temperature (°C); Twb, wet bulb temperature (°C).

**Figure 1:**
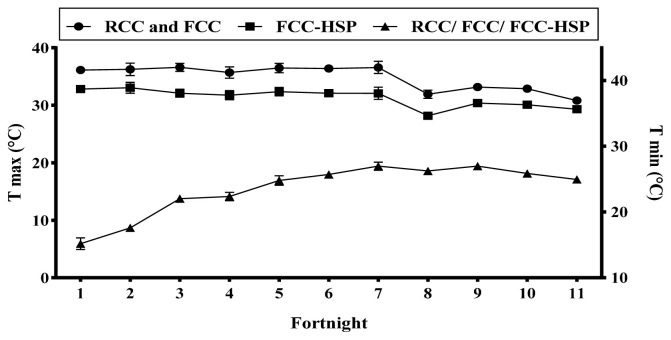
Fortnightly mean maximum temperature (T max) (°C) inside the sheds of restricted calf contact and fence line calf contact (RCC and FCC) and fence line calf contact and heat stress protection (FCC-HSP) groups and the fortnightly minimum temperature (T min) (°C) inside the sheds of fence line calf contact/fence line calf contact/fence line calf contact and heat stress protection (RCC/FCC/FCC-HSP) groups.

**Figure 2:**
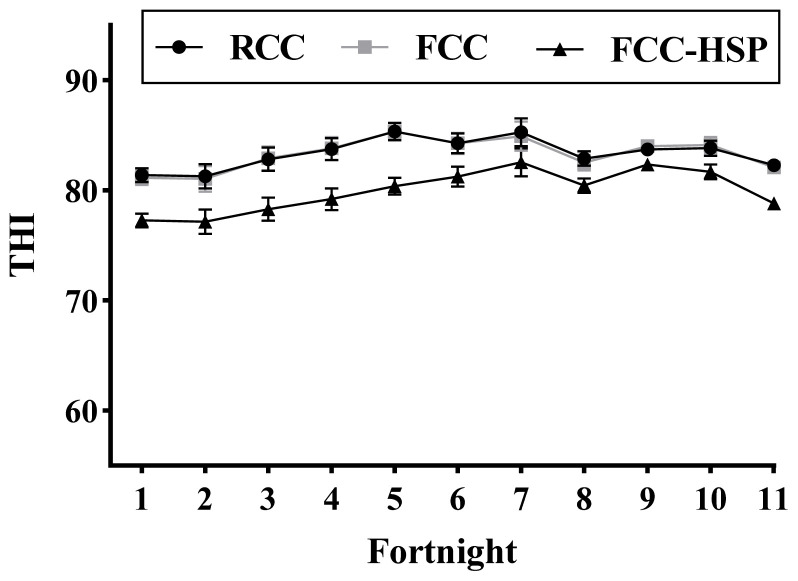
Fortnightly mean THI inside the sheds of restricted calf contact (RCC), fence line calf contact (FCC) and fence line calf contact and heat stress protection (FCC-HSP) groups.

Environmental heat stress on animals can be best expressed by THI [23], so THI was used to measure climatic stress on animals. Maximum and minimum fortnightly mean±standard error (SE) temperatures (°C) of various groups are presented in the Figure 1. Maximum and minimum temperature during the experimental period ranged 30.81°C to 36.60°C, 30.82°C to 36.62°C, and 29.31°C to 33.04°C in RCC, FCC and FCC-HSP, respectively. Calculated fortnightly mean±SE THI is presented in the Figure 2. During the experimental period (April to mid September) the average THI value ranged from 81.27±1.10 to 85.34±0.77, 81.05±1.15 to 85.34±0.82, and 77.16±1.22 to 82.55±1.26 in RCC, FCC and FCC-HSP, respectively.

### Measurement of physiological parameters

Physiological parameters recorded were rectal temperature (RT), pulse rate (PR), and respiration rate (RR). All parameters were recorded between 2.30 P.M to 3.00 P.M. RT was recorded using digital thermometer by keeping its bulb in contact to rectal mucosa for at least 2 min or till it beeped. PR was recorded from the coccygeal artery which is below the tail by finger and numbers of pulse in a minute were counted. RR was recorded by observing the flank movement of buffaloes, number of movements in a minute was recorded.

### Milk yield and composition

Daily milk yield was determined by weighing both the daily milk yield and the milk consumed by the calves, which was calculated by weighing the calves before and after each milking session. Daily a milk sample of approximately 20 mL was obtained from each buffalo, and its composition, including fat, protein, lactose, and solid-not-fat (SNF) content, was analyzed using an automatic milk analyzer machine (Lactoscan MCCW-V1, manufactured in Bulgaria).

### Enzyme-linked immunosorbent assay (ELISA) for plasma cortisol and prolactin

The collection of blood was done in morning before providing feed and water. Blood was collected from the jugular vein at fortnightly intervals in 9 mL BD vacutainers (Lithium Heparin coated). Afterward, the vacutainer tubes were gently rotated between the hands to aid in mixing the blood with the anticoagulant. Subsequently, the tubes were promptly placed in a refrigerator. To separate the plasma, the blood samples were centrifuged at 1,100 rpm for 15 minutes. The plasma obtained was then transferred to labelled storage vials and stored in a deep freezer (−20°C) for plasma cortisol and plasma prolactin measurements. Plasma cortisol and plasma prolactin were measured using ‘Bovine Cortisol ELISA Kit’ and ‘Bovine Prolactin ELISA Kit’ supplied by Bioassay Technology Laboratory, Jiaxing, Zhejiang, China.

### Statistical analysis

Milk yield, pulse rate, rectal temperature, respiration rate, cortisol and prolactin were compared using a mixed model (MIXED proc of SAS [PROC MIXED, SAS University Edition, SAS Institute Inc., Raleigh, NC, USA]). The model included treatment, time and their interaction as fixed effects, and the individual buffaloes included as random effects. Post hoc comparisons were performed with the pdiff option of SAS. Differences were considered statistically significant when p<0.05. Results are presented as lease squares means± standard error of the mean.

## RESULTS AND DISCUSSION

### Milk production

**Figure 3:**
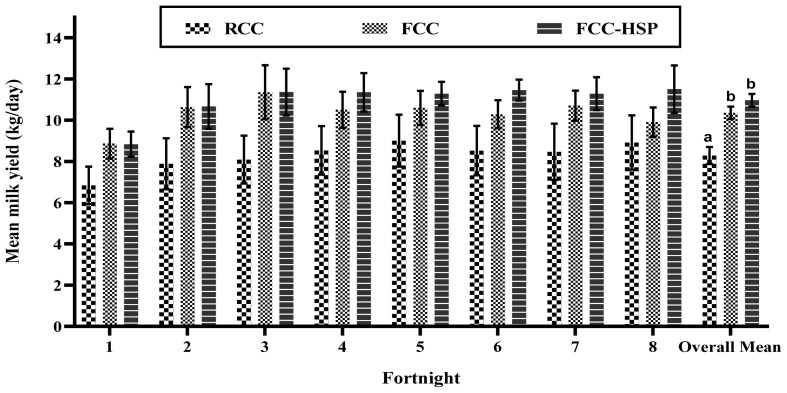
Mean daily milk yield (kg/d) of restricted calf contact (RCC), fence line calf contact (FCC) and fence line calf contact and heat stress protection (FCC-HSP) group of buffaloes at fortnight interval along with overall mean daily milk yield. The letters a and b indicate significant differences among groups, respectively (p<0.05).

Change in milk yield was seen between all the three groups from 6th day postpartum till 126th days of calving. Fortnight average mean±SE values of milka yields of various groups were calculated and are presented in Figure 3. No significant difference was seen in change in milk yields in each fortnight intervals between all the three groups. But a greater milk yield was seen in both the treatment groups compared to RCC group, and in the treatment groups a greater milk yield was seen in the FCC-HSP group than in the FCC group (Figure 3). Higher milk yield was reported by Choudhary et al [24] and Hassan et al [25] in FCC buffaloes as compared to restricted and weaned in Sahiwal and buffalo calves’ dams. Significant (p<0.05) difference was seen in overall milk yield between the RCC and treatment groups. But no significant difference was seen in overall milk yield between the treatment groups. The overall mean±SE milk yield till 120 days in RCC, FCC, and FCC-HSP groups was 8.29±0.41, 10.36± 0.30, and 10.97±0.31, respectively. This shows that on average 600 gm more milk was obtained in FCC-HSP than FCC group. Similar findings were reported by Kumar [11], Ahmad et al [18] and Savaliya et al [26] in Murrah, Nili-Ravi, Jaffarawadi buffaloes during summer season. They reported that when a time-controlled fan and fogging system is installed in the housing, milk yield increased as compared to control group with no modification in housing.

Overall mean±SE of various milk components is presented in Table 2. A significant difference in fat (%) and lactose (%) components of milk was seen between all the three groups. No significant difference was seen in protein (%) and SNF (%) component of milk between the groups. But Ahmad et al [18] reported increase in fat and protein (%) in case of the group in which heat stress ameliorative measures were provided. Choudhary et al [24] reported similar findings that FCC had no effect on protein and lactose content, but significant difference was seen in fat (%).

### Rectal temperature

RT is one of the indicators to represent the stress levels on animals. Fortnightly and overall means±SE RT of the animals are presented in the Table 3. There was a significant difference (p<0.05) between FCC-HSP and RCC/FCC groups. But no significant difference was seen between the RCC and FCC groups. The significant difference in RT in FCC-HSP group might be due to heat stress ameliorative measures employed in the shed. Gudev et al [8] reported higher RT in Bulgarian Murrah buffaloes with increase in THI levels because they were unable to maintain core body temperature. Further, no significant difference was seen within the groups in subsequent fortnights. Lakhani et al [27] also reported higher RT in Murrah buffaloes with higher THI levels. Rectal temperature of Nili-Ravi buffaloes and Murrah buffaloes reduced significantly in treatment group with microclimatic modification as compared to control group in summer season [5,16].

### Pulse rate and respiration rate

Fortnightly and overall mean±SE PR of the animals is presented in Table 4. Overall, there is a significant difference (p<0.05) in PR between all three groups. Mean PR during summer season was significantly (p<0.05) lower in FCC-HSP buffaloes compared to FCC and RCC buffaloes on all fortnights except on 2nd, 7th, and 8th fortnights where FCC-HSP buffaloes had the lowest PR followed by FCC and RCC buffaloes. The overall mean PR was also significantly (p<0.05) different among groups, with FCC-HSP (51.70± 0.35 beats/min) group having lowest PR followed by FCC (63.79±0.50 beats/min) and RCC (68.94±0.42 beats/min) group buffaloes. Increase in RR is one of the indicators of heat stress in animals. RR acts as characteristic physiological marker of heat and humidity stress in buffaloes [28]. Open mouth breathing is commonly seen in animals with excessive heat stress. Fortnightly and overall means±SE RR of the animals are presented in Table 5. There was significant (p< 0.05) difference in mean RR among the three groups of buffaloes, with FCC-HSP calves having a lower respiration rate followed by FCC and RCC group buffaloes on all fortnights except on 2nd, 3rd, 4th, and 5th fortnight where RR was significantly (p<0.05) higher in FCC-HSP group as compared to FCC and RCC group buffaloes. Overall, there is a significant difference (p<0.05) between all three groups. Higher temperatures cause cattle and buffaloes to breathe more quickly [29]. Lower PR in FCC-HSP might be due to cumulative effect of microclimatic modifications and the FCC. Manjari et al [28] also recorded higher PR in Tarai buffaloes when THI was more than 72. While Chaudhary et al [30] found no significant difference in PR of Surti buffaloes during hot dry, hot humid period than in comfort zone. These findings of the increased PR in RCC group buffaloes due to separation from calves agree with that of Newberry and Swanson [31]. Choudhary et al [24] and Enríquez et al [32] reported lower PR and RR in FCC group as compared to restricted contact group of mothers. Similarly, Sethi et al [33] recorded lower PR in Murrah buffaloes which were provided with shed and 2 showers during the day as compared to no shower group. While in a study when mist fans were provided in the shed the PR of Nili-ravi buffaloes reduced significantly as compared to no mist fan group of animals [5,18]. In a similar study by Yadav et al [16] similar findings were reported showing, reduced PR and RR of Murrah buffaloes in the shed provided with fans and foggers. Chaudhary et al [30] reported similar findings in Surti buffaloes, in which higher RR were recorded with increase in THI. Somagond et al [34] also reported similar findings in Murrah buffaloes, RR significantly reduced in buffaloes when treated feed was fed as compared to non-treated animals during the year when THI levels were more than 76. Das et al [5] reported that with modifications in the microclimatic conditions the RR significantly reduced in Nili-Ravi buffaloes.

### Plasma cortisol

Cortisol levels in the blood indicate the stress in animals. Fortnightly and overall mean±SE plasma cortisol levels of the animals are presented in the Table 6. Overall, there was a significant difference (p<0.05) between all three groups. But no significant difference was seen within the groups in all subsequent fortnights. In subsequent fortnights no significant difference between RCC and FCC groups but significant difference (p<0.05) was seen between FCC-HSP and RCC/FCC group. The fence line contact group offers an approach of calf separation that is progressive rather than abrupt, which is useful to minimize the stress. Similarly, Price et al [12] reported that cows and calves interacting along the fence line showed signs of adaptation to separation as they gradually became more independent of one another and spent less time there. Similar results were seen by Pérez-Torres et al [35] and Choudhary et al [24] in restricted calf and fence line contact groups where higher cortisol levels were seen in restricted contact group mothers. Separation of calves from mother is also a cause for increased stress [36]. A higher cortisol level in RCC and FCC groups than FCC-HSP indicates the level of stress on these groups. The lowest cortisol level in FCC-HSP group might be due to synergistic effect of fence line contact with calves and heat stress amelioration. Lower cortisol level indicates that heat stress ameliorative measures help in reducing stress on animals. Similar findings were reported by Yadav et al [16] in Murrah buffaloes, when fogger and fan were provided in the shed the cortisol levels reduced as compared to no fogger and fan group.

### Plasma prolactin

Prolactin levels in the blood are an indicator of heat stress in animals [37]. Fortnightly and overall mean±SE plasma prolactins of the animals are presented in the Table 7. Overall, there was a significant difference (p<0.05) between all three groups, with FCC-HSP buffaloes having a lower serum prolactin concentration followed by FCC and RCC group of buffaloes. Significant difference (p<0.05) was also seen within the groups in most of the subsequent fortnights. The increased prolactin levels in RCC and FCC then FCC-HSP group clearly show the effect of heat stress on these groups. In FCC-HSP group, the heat stress ameliorative measures helped in reducing the heat stress on animals thereby reduced prolactin levels. Yadav et al [16] reported similar findings in Murrah buffaloes with lowered prolactin levels in the treatment group provided with heat stress ameliorative measures (misting and wallowing) than in the control group. Similar findings were reported by Somagond et al [34] in Murrah buffaloes, in which higher levels of prolactin levels were reported when THI level was higher but when buffaloes were fed with treated feed the prolactin levels reduced. Prolactin levels in our study were higher as compared [16,34,37] to all other studies as calf contact in our study might have been responsible for the higher prolactin levels. Singh and Prakash [38] and Choudhary et al [24] have also reported higher prolactin levels in the buffaloes which were in calf contact compared to contact group.

## CONCLUSION

Fence line calf contact along with ameliorative measures reduced the harmful effects of heat stress on buffaloes by lowering the pulse rate, respiration rate, prolactin and cortisol levels and maintained the milk yield throughout the period of high THI. It was concluded that fence line mother-calf contact and suckling along with heat stress mitigation measures during summer season reduced the stress levels and remarkably improved the milk production in buffaloes.

## Figures and Tables

**Table 1:** Nutrient and ingredient composition (%) of concentrate mixture

Items	
Digestible crude protein (%)	17
Total digestible nutrients (%)	70
Energy (kcal/g)	2,470
Ingredients (%)	
Maize	35
Groundnut cake	18
Soya deoiled cake	19
Wheat bran	12
Deoiled rice bran	13
Mineral mixture	2
Common salt	1

**Table 2:** Mean milk composition of different groups of buffaloes during summer season

Parameters	RCC	FCC	FCC-HSP
Fat (%)	7.98±0.03^c^	7.33±0.03^b^	7.00±0.03^a^
Protein (%)	3.76±0.02	3.78±0.02	3.76±0.02
Lactose (%)	4.78±0.02^b^	4.69±0.02^a^	4.85±0.02^c^
Solid-not-fat (%)	9.78±0.02	9.74±0.02	9.74±0.02

The values are mean±standard error of eight observations on seven animals.

RCC, restricted calf contact; FCC, fence line calf contact; FCC-HSP, fence line calf contact and heat stress protection.

a–c The values with different superscripts within a row differed significantly (p<0.05) among treatment groups, respectively.

**Table 3:** Mean rectal temperature (°C) of different groups of buffaloes during experimental period

Fortnight	RCC	FCC	FCC-HSP
1	39.10^b^±0.12	38.85^ab^±0.11	38.56^a^±0.05
2	39.07^b^±0.06	39.01^b^±0.13	38.62^a^±0.03
3	39.16^b^±0.16	38.90^ab^±0.14	38.53^a^±0.05
4	38.87±0.14	38.94±0.15	38.61±0.05
5	39.13^b^±0.12	39.05^ab^±0.04	38.58^a^±0.03
6	38.97^b^±0.11	38.93^ab^±0.09	38.65^a^±0.04
7	39.07^b^±0.13	39.03^b^±0.11	38.58^a^±0.04
8	39.05^b^±0.09	38.79^b^±0.10	38.43^a^±0.04
Overall mean±standard error	39.04^b^±0.04	38.95^b^±0.03	38.55^a^±0.01

The values are mean±standard error of eight observations on seven animals.

RCC, restricted calf contact; FCC, fence line calf contact; FCC-HSP, fence line calf contact and heat stress protection.

a,b The values with different superscripts within a row differed significantly (p<0.05) among treatment groups, respectively.

**Table 4:** Mean pulse rate (beats/min) of different groups of buffaloes during experiment

Fortnight	RCC	FCC	FCC-HSP
1	67.57^b^±1.45	64.00^b^±1.84	53.29^a^±0.92
2	68.29^c^±1.49	62.71^b^±0.99	51.43^a^±0.78
3	69.14^b^±1.82	64.71^b^±2.08	52.14^a^±1.22
4	68.29^b^±1.57	63.29^b^±1.94	50.43^a^±1.19
5	65.86^b^±1.28	65.86^b^±1.14	53.14^a^±1.20
6	68.29^b^±0.94	64.86^b^±1.64	53.29^a^±1.02
7	69.86^c^±1.35	64.57^b^±1.53	51.43^a^±1.39
8	69.29^c^±1.17	62.57^b^±1.94	48.29^a^±0.84
Overall mean ±standard error	68.94^c^±0.42	63.79^b^±0.50	51.70^a^±0.35

The values are mean±standard error of eight observations on seven animals.

RCC, restricted calf contact; FCC, fence line calf contact; FCC-HSP, fence line calf contact and heat stress protection.

a–c The values with different superscripts within a row differed significantly (p<0.05) among treatment groups, respectively.

**Table 5:** Mean respiration rate (times/min) of different groups of buffaloes during experiment

Fortnight	RCC	FCC	FCC-HSP
1	37.86^c^±1.96	33.29^b^±0.68	22.29^a^±0.71
2	35.43^b^±0.48	35.29^b^±0.75	24.57^a^±1.19
3	37.71^b^±1.08	33.86^b^±1.12	22.71^a^±1.97
4	37.43^b^±2.03	32.86^b^±1.08	23.43^a^±1.81
5	37.29^b^±1.51	33.57^b^±1.48	23.29^a^±1.29
6	40.71^c^±1.70	34.57^b^±1.23	24.29^a^±1.55
7	37.86^c^±1.37	32.43^b^±1.41	21.43^a^±1.19
8	39.29^c^±1.19	34.14^b^±0.51	23.57^a^±1.09
Overall mean ±standard error	38.45^c^±0.45	33.40^b^±0.32	23.08^a^±0.4

The values are mean±standard error of eight observations on seven animals.

RCC, restricted calf contact; FCC, fence line calf contact; FCC-HSP, fence line calf contact and heat stress protection.

a–c The values with different superscripts within a row differed significantly (p<0.05) among treatment groups, respectively.

**Table 6:** Mean cortisol concentration (ng/mL) of different groups of buffaloes at fortnightly intervals

Fortnight	RCC	FCC	FCC-HSP
1	41.43^b,YZ^±0.85	39.59^b,XY^±1.21	34.64^a,Z^±0.78
2	39.11^b,XY^±0.87	36.71^b,XY^±1.15	30.72^a,XYZ^±0.94
3	38.12^b,XY^±0.81	37.16^b,XY^±0.67	28.76^a,WXY^±0.76
4	43.09^b,Z^±0.73	41.08^b,Y^±1.21	31.57^a,YZ^±0.88
5	41.08^b,YZ^±0.65	39.09^b,XY^±0.83	27.58^a,WX^±1.00
6	37.11^b,X^±0.95	35.04^b,X^±1.35	25.46^a,W^±1.21
7	39.01^b,XY^±0.50	36.18^b,X^±0.81	26.60^a,W^±0.70
8	41.39^b,YZ^±1.50	37.28^b,XY^±0.81	25.26^a,W^±0.53
Overall mean±standard error	40.04^c^±0.38	37.77^b^±0.43	28.82^a^±0.53

The values are mean±standard error of eight observations on seven animals.

RCC, restricted calf contact; FCC, fence line calf contact; FCC-HSP, fence line calf contact and heat stress protection.

a–c The values with different superscripts within a row differ (p<0.05).

W–Z The values with different superscripts within a column differ (p<0.05) .

**Table 7:** Mean prolactin concentration (ng/mL) of different groups of buffaloes at fortnightly intervals

Fortnight	RCC	FCC	FCC-HSP
1	295.49^a^±0.87	286.04^b^±0.94	231.79^c^±0.98
2	283.08^a^±0.95	280.93^a^±1.07	228.15^b^±0.89
3	273.09^a^±0.82	269.29^b^±0.61	212.83^c^±0.76
4	264.16^a^±0.63	261.17^a^±1.18	204.10^b^±0.63
5	261.35^a^±1.05	254.68^b^±1.38	195.46^c^±0.74
6	252.34^a^±0.85	252.98^a^±1.07	187.99^b^±0.84
7	245.06^a^±1.14	239.29^b^±0.69	181.88^c^±1.04
8	225.61^a^±2.01	221.53^a^±0.79	174.82^b^±0.59
Overall mean ±standard error	262.52^a^±3.03	258.24^b^±2.92	202.13^c^±2.87

The values are LS mean±standard error of eight observations on seven animals.

RCC, restricted calf contact; FCC, fence line calf contact; FCC-HSP, fence line calf contact and heat stress protection.

a–c The values with different superscripts within a row differed significantly (p<0.05) among treatment groups, respectively.
